# Hospital Meetings

**Published:** 1905-02-04

**Authors:** 


### HOSPITAL MEETINGS.

### THE GERMAN HOSPITAL.

THE 60th annual meeting of the German Hospital was held on Thursday, January 26th, Mr. Arthur J. Allen presiding.

The Chairman said the reports were on the whole extremely satisfactory. The hospital had, however, sustained a great loss in the death of H.R H. the Duke of Cambridge, who had been for close upon 54 years President of the hospital, and during that time had shown deep personal interest in its welfare, while on 25 occasions he had presided at the annual dinner on behalf of the funds. A slight departure from the ordinary methods of analysis of expenditure had been adopted in the report for 1904, in which the average cost of each bed occupied was given, in addition to that of each in-patient, and each in-patient per week. The figures would be found to compare very favourably with those of other London hospitals. A powerful searchlight was at the present time turned upon all hospitals, and every trouble was taken to discover whether they could be conducted on improved methods. The German hospital, however, had nothing to fear from this investigation, and the greater the interest shown by his Majesty the King and H.R.H. the Prince of Wales in hospital administration the better it would be. After alluding to an approaching meeting with the honorary officers of King Edward's Hospital Fund, the chairman said the hospital owed an enormous sum to the treasurer, Baron von Schröder, whose absence he regretted; the Committee wished to express their heartfelt gratitude to him for his generous assistance in this way. Out of respect to the memory of their late president the annual dinner was not held last year, but the stewards, who were already at work, continued their kind efforts on behalf of the hospital, and succeeded in getting together the large amount of £3,184 15s. 5d., a sum only £20 less than that of the previous year, while there was a saving of some £250 in expenses. The Committee had several much-needed developments in view, which they were anxious to carry out as soon as the necessary funds were forthcoming. Among others were—(1) the addition of outside staircases, to afford means of escape for patients and staff in case of fire; (2) the provision of open-air balconies; (3) the enlargement of the out-patients' department, the accommodation in which was altogether inadequate for the increasing numbers, the attendances last year having been 9,932 more than in the previous year. Until, however, the present heavy debt of £5,908 18s. 3d. was liquidated the Committee did not feel justified in embarking upon fresh capital expenditure, except for the addition of outside staircases, which they considered should be erected at once; they therefore made a renewed earnest appeal for increased support, more particularly from the English and Jewish communities, in recognition of the extensive help which the hospital afforded to their own sick poor.

The report and accounts were adopted. It was resolved to ask the Austrian and Russian Ambassadors respectively to accept the office of vice-president; and, other business having been transacted, a vote of thanks to the chairman closed the proceedings.

### GENERAL LYING-IN HOSPITAL, YORK ROAD.

#### REORGANISATION OF THE TRAINING SCHOOL.

THE annual meeting of Governors of the General Lying-in Hospital, York Road, Lambeth, was held on Wednesday, January 25th, Lord Saye and Sele in the chair. The report presented opened with the remark that the past year had been full of grave anxiety to the Committee of Management. The Midwives Act had thrown great responsibility, as well as great opportunities for usefulness, upon institutions of this character, and after full consideration the committee had determined to develop their training school for midwives and monthly nurses, so as to embrace not only the in-patient but also the out-patient department. The new regulations came into force in July last, since which time 66 midwives and one nurse had been under training in the district, in addition to 33 midwives and 94 nurses who had received their training in the wards of the hospital. Notwithstanding this extension, applications from candidates continued to be far in excess of the number that could be received. A suitable building opposite the hospital had been acquired as a residential home to accommodate the midwives and nurses under training. Through the kindness of Dr. and Mrs. Boxall and their friends the home was furnished free of expense to the hospital, and the committee wished to take the opportunity of expressing their deep sense of obligation not only for this but for the reorganisation of the training school, which was carried out largely through the exertions of Dr. Boxall, who had also established a valuable midwifery scholarship. A promise of extension of lease for a long period of years had been obtained from the freeholders of the hospital site, which would enable the committee to carry out a long contemplated scheme of enlargement by the erection of a new storey, where more suitable accommodation for the nurses and servants would be possible. The existing building had been adapted from time to time, but such an enlargement, the committee were advised, was now absolutely necessary. The interest on the outlay would be defrayed by the addisional fees obtained from the school, but for the outlay itself the committee earnestly trusted in the supporters to prevent any undue encroachment upon accumulated capital. There had been a satisfactory increase in subscriptions, and a still more satisfactory decrease in excess of expenditure over income—viz., £90, as compared with £363 in the previous year. It had been found necessary to close the Convalescent Home at Woking.

The report and accounts having been adopted, Dr. Cullingworth congratulated the committee on the achievements of the past year, remarking that the medical report presented was most satisfactory and the medical results beyond reproach. He would have liked a somewhat more detailed report as to the extension of training to the externe district, this being an entirely new departure, of which it would be well to have some permanent record as to the exact scheme adopted.

Lord Lister having been unanimously re-elected as president for the ensuing year, the usual votes of thanks closed the proceedings.

### THE ITALIAN HOSPITAL.

The annual meeting of the governors of the Italian Hospital was held last Saturday, Signor F. Carignani di Novoli Italian Chargé d'Affaires taking the chair. The report for 1904 showed that during the year there were 538 in-patients and 11,761 out-patients. Unfortunately the accounts for the year showed a decrease in the donations and subscriptions. It was pointed out that but for a sum of £635 received from the Charity Committee of the Italian Chamber of Commerce as the result of entertainments given at the Italian Exhibition, the year's accounts would have shown a considerable deficit. The report having been adopted, Dr. Castaneda moved: "That the dutiful and sincere thanks of the governors and committee of management be and hereby are tendered with the greatest respect to his Royal Highness, the President of King Edward's Hospital Fund for London, for the yearly grant of £500 awarded from the Fund to keep open 10 beds." Lord Edmund Talbot, in seconding the motion, said that the public as a whole were aware of the work done by his Royal Highness in this way, but only those who were more intimately connected with hospital work knew how real and personal was the interest he took in it. The motion was adopted. In acknowledging a vote of thanks at the end of the meeting, Signor Carignani di Novoli said that he was in a position to assure those present that the King and Queen of Italy took a keen interest in the hospital

